# Mapping the Space of Genomic Signatures

**DOI:** 10.1371/journal.pone.0119815

**Published:** 2015-05-22

**Authors:** Lila Kari, Kathleen A. Hill, Abu S. Sayem, Rallis Karamichalis, Nathaniel Bryans, Katelyn Davis, Nikesh S. Dattani

**Affiliations:** 1 Department of Computer Science, University of Western Ontario, London, Ontario, Canada; 2 Department of Biology, University of Western Ontario, London, Ontario, Canada; 3 Physical and Theoretical Chemistry Laboratory, Department of Chemistry, Oxford University, Oxford, United Kingdom; University of Veterinary Medicine Hanover, GERMANY

## Abstract

We propose a computational method to measure and visualize interrelationships among any number of DNA sequences allowing, for example, the examination of hundreds or thousands of complete mitochondrial genomes. An "image distance" is computed for each pair of graphical representations of DNA sequences, and the distances are visualized as a Molecular Distance Map: Each point on the map represents a DNA sequence, and the spatial proximity between any two points reflects the degree of structural similarity between the corresponding sequences. The graphical representation of DNA sequences utilized, Chaos Game Representation (CGR), is genome- and species-specific and can thus act as a genomic signature. Consequently, Molecular Distance Maps could inform species identification, taxonomic classifications and, to a certain extent, evolutionary history. The image distance employed, Structural Dissimilarity Index (DSSIM), implicitly compares the occurrences of oligomers of length up to *k* (herein *k* = 9) in DNA sequences. We computed DSSIM distances for more than 5 million pairs of complete mitochondrial genomes, and used Multi-Dimensional Scaling (MDS) to obtain Molecular Distance Maps that visually display the sequence relatedness in various subsets, at different taxonomic levels. This general-purpose method does not require DNA sequence alignment and can thus be used to compare similar or vastly different DNA sequences, genomic or computer-generated, of the same or different lengths. We illustrate potential uses of this approach by applying it to several taxonomic subsets: phylum Vertebrata, (super)kingdom Protista, classes Amphibia-Insecta-Mammalia, class Amphibia, and order Primates. This analysis of an extensive dataset confirms that the oligomer composition of full mtDNA sequences can be a source of taxonomic information. This method also correctly finds the mtDNA sequences most closely related to that of the anatomically modern human (the Neanderthal, the Denisovan, and the chimp), and that the sequence most different from it in this dataset belongs to a cucumber.

## Introduction

Even though every year biologists discover and classify thousands of new species, it is estimated that as many as 86% of existing species on Earth and 91% of species in the oceans have not yet been classified and catalogued, [1]. In the absence of a universal quantitative method to identify species’ relationships, information for species classification has to be gleaned and combined from several sources, e.g., morphological, sequence-alignment-based phylogenetic anaylsis, and non-alignment-based molecular information.

We propose a computational process that outputs, for any given dataset of DNA sequences, a concurrent display of the structural similarities among all sequences in the dataset. This is obtained by first computing an “image distance” for each pair of graphical representations of DNA sequences, and then visualizing the resulting interrelationships in a two-dimensional plane. The result of applying this method to a collection of DNA sequences is an easily interpretable *Molecular Distance Map* wherein sequences are represented by points in a common Euclidean plane, and the spatial distance between any two points reflects the differences in their subsequence composition.

The graphical representation we use is *Chaos Game Representation* (CGR) of DNA sequences, [2, 3], that simultaneously displays all subsequence frequencies of a given DNA sequence as a visual pattern. CGR has a remarkable ability to differentiate between genetic sequences belonging to different species, and has thus been proposed as a *genomic signature*. Due to this characteristic, a Molecular Distance Map of a collection of genetic sequences may allow inferrences of relationships between the corresponding species.

Concretely, to compute and visually display relationships within a given set *S* = {*s*_1_, *s*_2_, …, *s*_*n*_} of *n* DNA sequences, we propose a computational process that uses:

(i) *Chaos Game Representation* (CGR), to graphically represent all subsequences of a DNA sequence *s*_*i*_, 1 ≤ *i* ≤ *n*, as pixels of one image, denoted by *c*_*i*_;

(ii) *Structural Dissimilarity Index* (DSSIM), an “image-distance” measure, to compute the pairwise distances Δ(*i*, *j*), 1 ≤ *i*, *j* ≤ *n*, for each pair of CGR images (*c*_*i*_, *c*_*j*_), and to produce a distance matrix;

(iii) *Multi-Dimensional Scaling* (MDS), an information visualization technique that takes as input the distance matrix and outputs a Molecular Distance Map in 2D, wherein each plotted point *p*_*i*_ with coordinates (*x*_*i*_, *y*_*i*_) represents the DNA sequence *s*_*i*_ whose CGR image is *c*_*i*_. The position of the point *p*_*i*_ in the map, relative to all the other points *p*_*j*_, reflects the distances between the DNA sequence *s*_*i*_ and the other DNA sequences *s*_*j*_ in the dataset.

We apply this method to analyze and visualize several different taxonomic subsets of a dataset of 3,176 complete mtDNA sequences: phylum Vertebrata, (super)kingdom Protista, classes Amphibia-Insecta-Mammalia, class Amphibia only, and order Primates. We illustrate the usability of this approach by discussing, e.g., the placement of the genus *Polypterus* within phylum Vertebrata, of the unclassified organism *Haemoproteus* sp. jb1.JA27 within the (super)kingdom Protista, and the placement of the family Tarsiidae within the order Primates. We also provide an interactive web tool, *MoD Map* (*Mo*lecular *D*istance *Map*), that allows an in-depth exploration of all Molecular Distance Maps in the paper, complete with zoom-in features, search options, and easily accessible additional information for each sequence-representing point (called hereafter sequence-point).

Overall, this method groups mtDNA sequences in correct taxonomic groups, from the kingdom level down to the order and family level. These results are of interest both because of the size of the dataset and because this information was extracted from DNA sequences that normally would not be considered in alignment-based comparison methods. Our analysis confirms that sequence composition (presence or absence of oligomers) contains taxonomic information that could be relevant to species identification, taxonomic classification, and identification of large evolutionary lineages. Last but not least, the appeal of this method lies in its simplicity, robustness, and generality, whereby exactly the same measuring tape can automatically yield meaningful measurements between non-specific DNA sequences of species as distant as those of the anatomically modern human and a cucumber, and as close as those of the anatomically modern human and the Neanderthal.

## Methods

A CGR [2, 3] associates an image to each DNA sequence as follows. Begin with a unit square with corners labelled *A, C, G,* and *T*, clockwise starting from the bottom-left corner. The first point of any CGR plot is the center of the square. To plot the CGR corresponding to a given DNA sequence, start reading the letters of the sequence from left to right, one by one. The point corresponding to the first letter is the point plotted in the middle of the segment determined by the center of the square and the corner labelled by the first letter. For example, if the center of the square is labelled “O” and the first letter of the sequence is “A”, then the point of the plot corresponding to the first “A” is the point situated halfway between O and the corner A. Subsequent letters are plotted iteratively as the middle point between the previously-drawn-point and the corner labelled by the letter currently being read.

**Fig 1:**
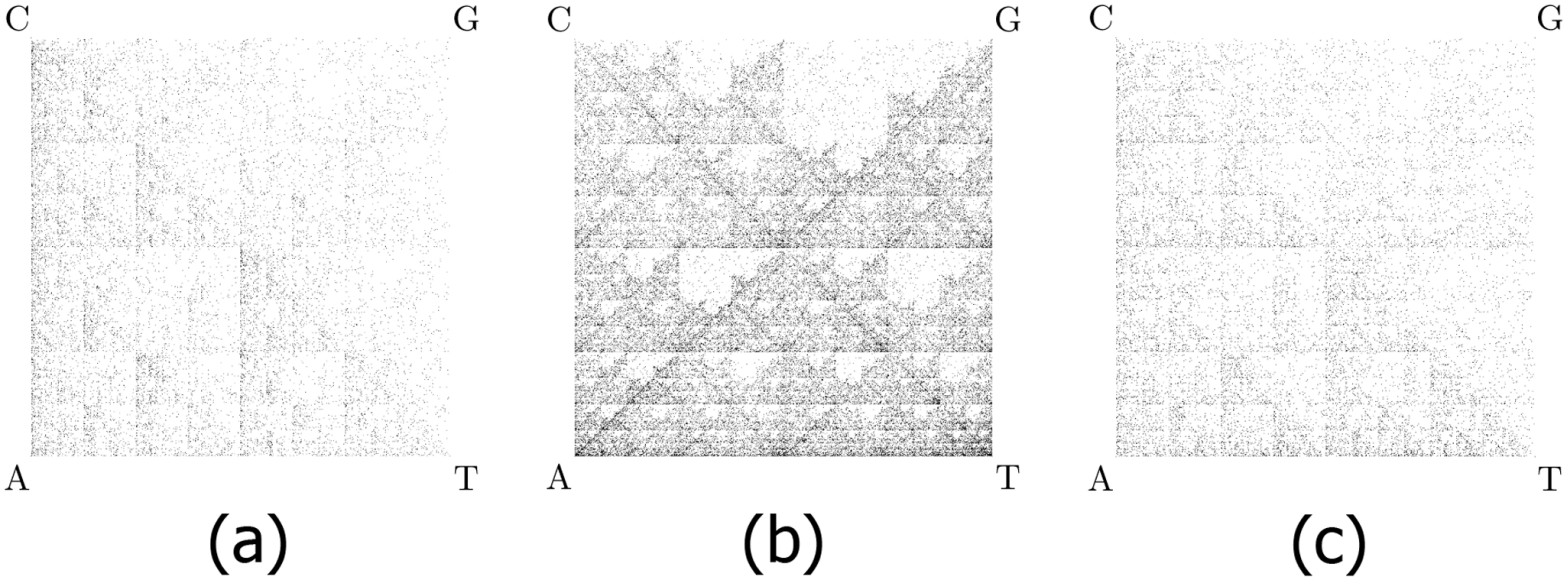
CGR images for three DNA sequences. (a) *Homo sapiens sapiens* mtDNA, 16,569 bp; (b) *Homo sapiens sapiens* chromosome 11, beta-globin region, 73,308 bp; (c) *Polypterus endlicherii* (fish) mtDNA, 16,632 bp. Observe that chromosomal and mitochondrial DNA from the same species can display different patterns, and also that mtDNA of different species may display visually similar patterns that are however sufficiently different as to be computationally distinguishable.

CGR images of genetic DNA sequences originating from various species show rich fractal patterns containing various motifs such as squares, parallel lines, rectangles, triangles and diagonal crosses, see, e.g., Fig. 1. CGRs of genomic DNA sequences have been shown to be genome- and species-specific, [2–8]. Thus, sequences chosen from each genome as a basis for computing “distances” between genomes do not need to have any relation with one another from the point of view of their position or information content. In addition, this graphical representation facilitates easy visual recognition of global string-usage characteristics: Prominent diagonals indicate purine or pyrimidine runs, sparseness in the upper half indicates low G+C content, etc., see for example [6].

If the generated CGR image has a resolution of 2^*k*^ × 2^*k*^ pixels, then every pixel represents a distinct DNA subsequence of length *k*: A pixel is black if the subsequence it represents occurs in the DNA sequence, otherwise it is white. In this paper, for the CGR images of all 3,176 complete mtDNA sequences in our dataset, we used the value *k* = 9, that is, occurrences of subsequences of lengths up to 9 were being taken into consideration. In general, a length of DNA sequence of about 4,000 bp is necessary to obtain a well-defined CGR, but a length of 2,000 bp can sometimes give a good approximation, [2]. In our case, we used the full length of all analyzed mtDNA sequences, which ranged from 288 bp to 1,555,935 bp, with an average of 28,000 bp.

Other visualizations of genetic data include the 2D rectangular walk [9] and methods similar to it in [10, 11], vector walk [12], cell [13], vertical vector [14], Huffman coding [15], and colorsquare [16] methods. Three-dimensional representations of DNA sequences include the tetrahedron [17], 3D-vector [18], and trinucleotide curve [19] methods. Among these visualization methods, CGR images arguably provide the most immediately comprehensible “signature” of a DNA sequence and a desirable genome-specificity, [2, 7]. In addition, the images produced using CGR are easy to compare, visually and computationally. Coloured versions of CGR, wherein the colour of a point corresponds to the frequency of the corresponding oligomer in the given DNA sequence (from red for high frequency, to blue for no occurrences) have also been proposed [20, 21].

Note that other alignment-free methods have been used for phylogenetic analysis of DNA strings, such as computing the Euclidean distance between frequencies of *k*-mers (*k* ≤ 5) for the analysis of 125 GenBank DNA sequences from 20 bird species and the American alligator, [22]. Another study, [23], analyzed 459 dsDNA bacteriophage genomes and compared them with their host genomes to infer host-phage relationships, by computing Euclidean distances between frequencies of *k*-mers for *k* = 4. In [24], 75 complete HIV genome sequences were compared using the Euclidean distance between frequencies of 6-mers (*k* = 6), in order to group them into subtypes. In [25], 27 microbial genomes were analyzed to find implications of 4-mer frequencies (*k* = 4) on their evolutionary relationships. In [26], 20 mammalian complete mtDNA sequences were analyzed using a so-called “similarity metric”. Our method uses a larger dataset (3,176 complete mtDNA sequences), an “image distance” measure that was designed to capture structural similarities between images, as well as a value of *k* = 9.

Structural Similarity (SSIM) index is an image similarity index used in the context of image processing and computer vision to compare two images from the point of view of their structural similarities [27]. SSIM combines three parameters—luminance distortion, contrast distortion, and linear correlation—and was designed to perform similarly to the human visual system, which is highly adapted to extract structural information. Originally, SSIM was defined as a similarity measure *s*(*A*, *B*) whose theoretical range between two images *A* and *B* is [−1, 1] where a high value amounts to close relatedness. We use a related *DSSIM distance* Δ(*A*, *B*) = 1 − *s*(*A*, *B*) ∈ [0, 2], with the distance being 0 between two identical images, 1 for example between a black image and a white image, and 2 if the two images are negatively correlated, that is, Δ(*A*, *B*) = 2 if and only if every pixel of image *A* has the inverted value of the corresponding pixel in image *B* while both images have the same luminance (brightness). For our particular dataset of genetic CGR images, almost all (over 5 million) distances are between 0 and 1, with only half a dozen exceptions of distances between 1 and 1.0033.

MDS has been used for the visualization of data relatedness based on distance matrices in various fields such as cognitive science, information science, psychometrics, marketing, ecology, social science, and other areas of study [28]. MDS takes as input a distance matrix containing the pairwise distances between *n* given items and outputs a two-dimensional map wherein each item is represented by a point, and the spatial distances between points reflect the distances between the corresponding items in the distance matrix. Notable examples of molecular biology studies that used MDS are [29] (where it was used for the analysis of geographic genetic distributions of some natural populations), [30] (where it was used to provide a graphical summary of the distances among CO1 genes from various species), and [31] (where it was used to analyze and visualize relationships within collections of phylogenetic trees).

Classical MDS, which we use in this paper, receives as input an *n* × *n* distance matrix (Δ(*i*, *j*))_1 ≤ *i*,*j* ≤ *n*_ of the pairwise distances between any two items in the set. The output of classical MDS consists of *n* points in a *q*-dimensional space whose pairwise spatial (Euclidean) distances are a linear function of the distances between the corresponding items in the input distance matrix. More precisely, MDS will return *n* points *p*_1_, *p*_2_, …, *p*_*n*_ ∈ ℝ^*q*^ such that *d*(*i*, *j*) = ∣∣*p*_*i*_ − *p*_*j*_∣∣ ≈ *f*(Δ(*i*, *j*)) for all *i*, *j* ∈ {1, …, *n*} where *d*(*i*, *j*) is the spatial distance between the points *p*_*i*_ and *p*_*j*_, and *f* is a function linear in Δ(*i*, *j*). Here, *q* can be at most *n* − 1 and the points are recovered from the eigenvalues and eigenvectors of the input *n* × *n* distance matrix. If we choose *q* = 2 (respectively *q* = 3), the result of classical MDS is an approximation of the original (*n* − 1)-dimensional space as a two- (respectively three-) dimensional map.

In this paper all Molecular Distance Maps consist of coloured points, wherein each point represents an mtDNA sequence from the dataset. Each mtDNA sequence is assigned a unique numerical identifier retained in all analyses, e.g., #1321 is the identifier for the *Homo sapiens sapiens* mitochondrial genome. The colour assigned to a sequence-point may however vary from map to map, and it depends on the taxon assigned to the point in a particular Molecular Distance Map and the colour associated to that taxon in that map. For consistency, all maps are scaled so that the *x*- and the *y*-coordinates always span the interval [−1, 1]. The formula used for scaling is $x_{\text{sca}}=2\cdot (\frac{x-x_{\text{min}}}{x_{\text{max}}-x_{\text{min}}})-1$, $y_{\text{sca}}=2\cdot (\frac{y-y_{\text{min}}}{y_{\text{max}}-y_{\text{min}}})-1$, where *x*_min_ and *x*_max_ are the minimum and maximum of the *x*-coordinates of all the points in the original map, and similarly for *y*_min_ and *y*_max_.

Each Molecular Distance Map has some error, that is, the spatial distances *d*_*i*, *j*_ are not exactly the same as *f*(Δ(*i*, *j*)). When using the same dataset, the error is in general lower for an MDS map in a higher-dimensional space. The *Stress-1* (Kruskal stress, [32]), is defined in our case as

$$\begin{array}{c} \text{Stress-1}=\sigma_{1}=\sqrt{\frac{\Sigma_{i<j}{\left[f\left(\Delta \left(i,j\right)\right)-d_{i,j}\right]}^{2}}{\Sigma_{i<j}d_{i,j}^{2}}} \end{array}$$

where the summations extend over all the sequences considered for a given map, and *f*(Δ(*i*, *j*)) = *a* × Δ(*i*, *j*) + *b* is a linear function whose parameters *a*, *b* ∈ ℝ are determined by linear regression for each subset and corresponding Molecular Distance Map. A benchmark that is often used to assess MDS results is that *Stress-1* should be in the range [0, 0.20], see [32].

The dataset consists of the entire collection of complete mitochondrial DNA sequences from NCBI as of 12 July, 2012. This dataset consists of 3,176 complete mtDNA sequences, namely 79 protists, 111 fungi, 283 plants, and 2,703 animals. This collection of mitochondrial genomes has a great breadth of species across taxonomic categories and great depth of species coverage in certain taxonomic categories. For example, we compare sequences at every rank of taxonomy, with some pairs being different at as high as the (super)kingdom level, and some pairs of sequences being from the exact same species, as in the case of *Silene conica* for which our dataset contains the sequences of 140 different mitochondrial chromosomes [33]. The prokaryotic origins and evolutionary history of mitochondrial genomes have long been extensively studied, which will allow comparison of our results with known relatedness of species. Lastly, this genome dataset permits testing of both recent and deep rooted species relationships, providing fine resolution of species differences.

The creation of the datasets, acquisition of data from NCBI’s GenBank, generation of the CGR images, calculation of the distance matrix, and calculation of the Molecular Distance Maps using MDS, were all done (and can be tested with) the free open-source MATLAB program OpenMDM [34]. This program makes use of an open source MATLAB program for SSIM, [27], and MATLAB’s built-in MDS function. The interactive web tool *MoD Map*, [35], allows an in-depth exploration and navigation of the Molecular Distance Maps in this paper. When using the web tool *MoD Map*, clicking on the “Draw MoD Map” button allows the selection of any of the five maps presented in the paper, each with features such as zoom-in and search by scientific name of the species or the NCBI accession number of its mtDNA. On any given Molecular Distance Map, clicking on a sequence-point displays its full mtDNA sequence information such as its unique identifier in this analysis, NCBI accession number, scientific name, common name, length of mtDNA sequence, taxonomy, CGR image, as well as a link to the corresponding NCBI entry. Clicking on the “From here” and “To here” buttons displays the image distance between the CGR images of two selected sequence-points, as a number between 0 and 1.

## Results and Discussion

The Molecular Distance Maps we analyzed, of several different taxonomic subsets (phylum Vertebrata, (super)kingdom Protista, classes Amphibia-Insecta-Mammalia, class Amphibia only, and order Primates), confirm that the presence or absence of oligomers in mtDNA sequences may contain information that is relevant to taxonomic classifications. These results are relevant because they are the output of a method that bypasses the need of sequence alignment and uses as input DNA sequences that would not generally be considered by other, alignment-based, methods. The main contributions of the paper are the following:

- The use of an “image distance” (designed to detect structural similarities between images) to compare the graphic signatures of two DNA sequences. For any given *k*, this distance simultaneously compares the occurrences of all subsequences of length up to *k* of the two sequences. In all computations of this paper we use *k* = 9. This image distance (with parameter set to *k* = 9) is highly sensitive and succeeds to successfully group hundreds of CGRs that are visually similar, such as the ones in Fig. 1(a) and Fig. 1(c), into correct taxonomic categories.
- The use of an information visualization technique to display the results as easily interpretable Molecular Distance Maps, wherein the spatial position of each sequence-point in relation to all other sequence-points is quantitatively significant. This is augmented by an interactive web tool which allows an in-depth exploration of the Molecular Distance Maps in this paper, with features such as zoom-in, search by scientific name or NCBI accession number, and quick access to complete information for each of the full mtDNA sequences in the map.
- A method that is general-purpose, simple, computationally efficient and scalable. Since the compared sequences need not be homologous or of the same length, this method can be used to provide comparisons among any number of completely different DNA sequences: within the genome of an individual, across genomes within a single species, between genomes within a taxonomic category, and across taxa.
- The use of a large dataset of 3,176 complete mitochondrial DNA sequences.
- An illustration of potential uses of this approach by the discussion of several case studies such as the placement of the genus *Polypterus* within phylum Vertebrata, of the unclassified organism *Haemoproteus* sp. jb1.JA27 (#1466) within the (super)kingdom Protista, and the placement of the family Tarsiidae within the order Primates.

This method could complement information obtained by using DNA barcodes [30] and Klee diagrams [36], since it is applicable to cases where barcodes may have limited effectiveness: plants and fungi for which different barcoding regions have to be used [37–39]; protists where multiple loci are generally needed to distinguish between species [40]; prokaryotes [41]; and artificial, computer-generated, DNA sequences. This method may also complement other taxonomic analyses by bringing in additional information gleaned from comparisons of non-homologous and non-coding sequences.

**Fig 2:**
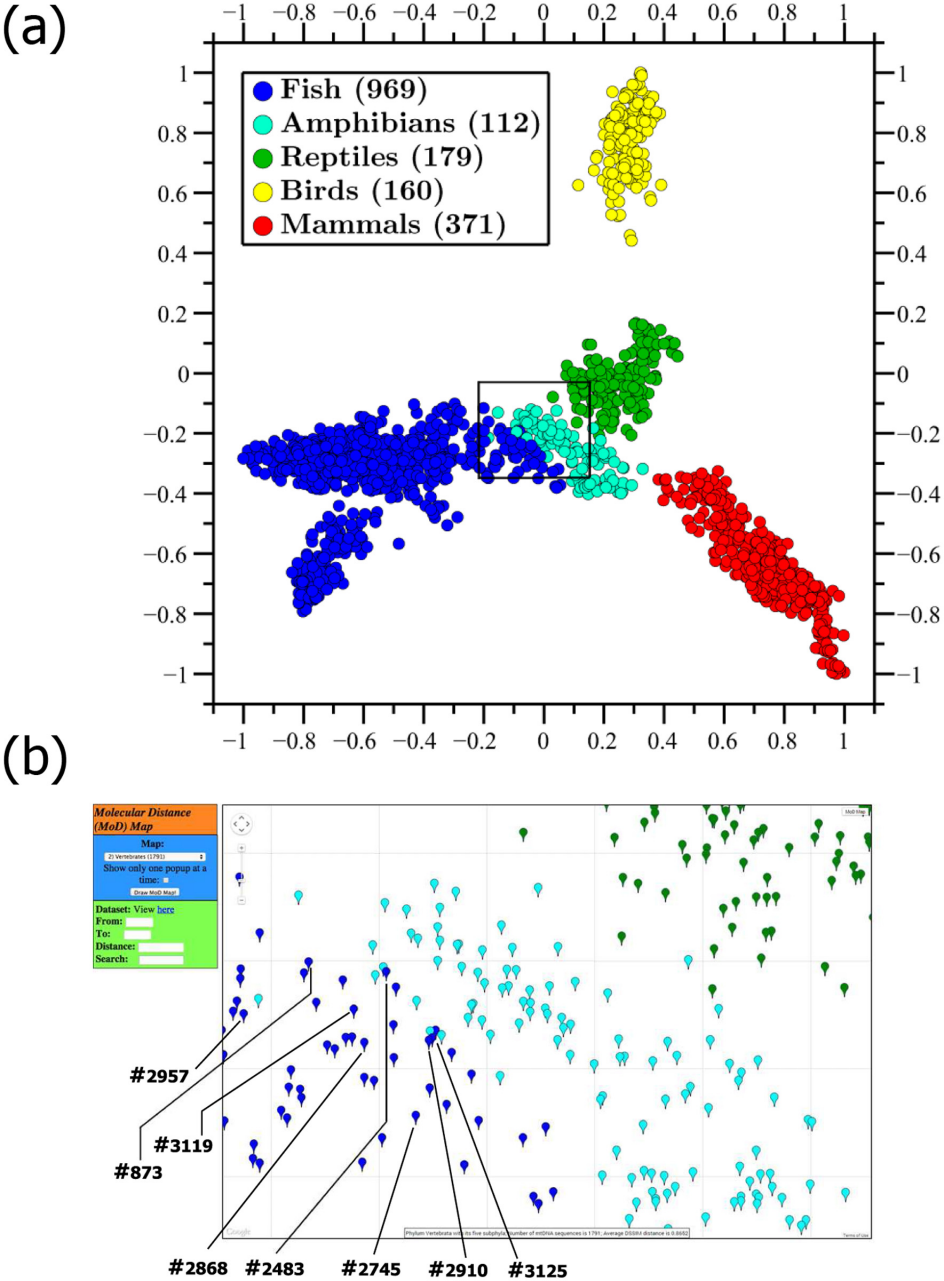
Molecular Distance Map of phylum Vertebrata (excluding the 5 represented jawless vertebrates), with its five subphyla. (a) This Molecular Distance Map comprises 1,791 mtDNA sequences, the average DSSIM distance is 0.8652, and the MDS *Stress-1* is 0.12. Fish species bordering amphibians include fish with primitive pairs of lungs (*Polypterus ornatipinnis* #3125, *Polypterus senegalus* #2868), a fish who can breathe atmospheric air using a pair of lungs (*Erpetoichthys calabaricus* #2745), a toadfish (*Porichtys myriaster* #2483), and all four represented lungfish (*Protopterus aethiopicus* #873, *Lepidosiren paradoxa* #2910, *Neoceratodus forsteri* #2957, *Protopterus doloi* #3119). Note that the question of whether species of the genus *Polypterus* are fish or amphibians has been discussed extensively for hundreds of years. Note also that gaps and spaces in clusters, in this and other maps, may be due to sampling bias. (b) Screenshot of the zoomed-in rectangular region outlined in Fig. 2(a), obtained using the interactive web tool *MoD Map* [35].

An example of the CGR/DSSIM/MDS approach is the Molecular Distance Map in Fig. 2 which depicts the complete mitochondrial DNA sequences of all 1,791 jawed vertebrates in our dataset. (In the legends of all Molecular Distance Maps in this paper, the number of represented mtDNA sequences in each category is listed in paranthesis after the category name.) Note that the position of each point in a map is determined by *all* the distances between the sequence it represents and the other sequences in the dataset. In the case of Fig. 2, the position of each sequence-point is determined by the 1,790 numerical distances between its sequence and all the other vertebrate mtDNA sequences in that dataset.

Observe that all five different subphyla of jawed vertebrates are separated in non-overlapping clusters, with very few exceptions. Examples of fish species bordering or slightly mixed with the amphibian cluster include *Polypterus ornatipinnis* (#3125, ornate bichir), *Polypterus senegalus* (#2868, Senegal bichir), both with primitive pairs of lungs; *Erpetoichthys calabaricus* (#2745, reedfish) who can breathe atmospheric air using a pair of lungs; and *Porichtys myriaster* (#2483, specklefish midshipman) a toadfish of the order Batrachoidiformes. It is noteworthy that the question of whether species of the *Polypterus* genus are fish or amphibians has been discussed extensively for hundreds of years [42]. Interestingly, all four represented lungfish (a.k.a. salamanderfish), are also bordering the amphibian cluster: *Protopterus aethiopicus* (#873, marbled lungfish), *Lepidosiren paradoxa* (#2910, South American lungfish), *Neoceratodus forsteri* (#2957, Australian lungfish), *Protopterus doloi* (#3119, spotted African lungfish). In answer to the hypothesis in [22] regarding the diversity of signatures across vertebrates, we note that in Fig. 2 the avian mtDNA signatures cluster neither with the mammals nor with the reptiles, and form a completely separate cluster of their own (albeit closer to reptiles than to mammals).

**Fig 3:**
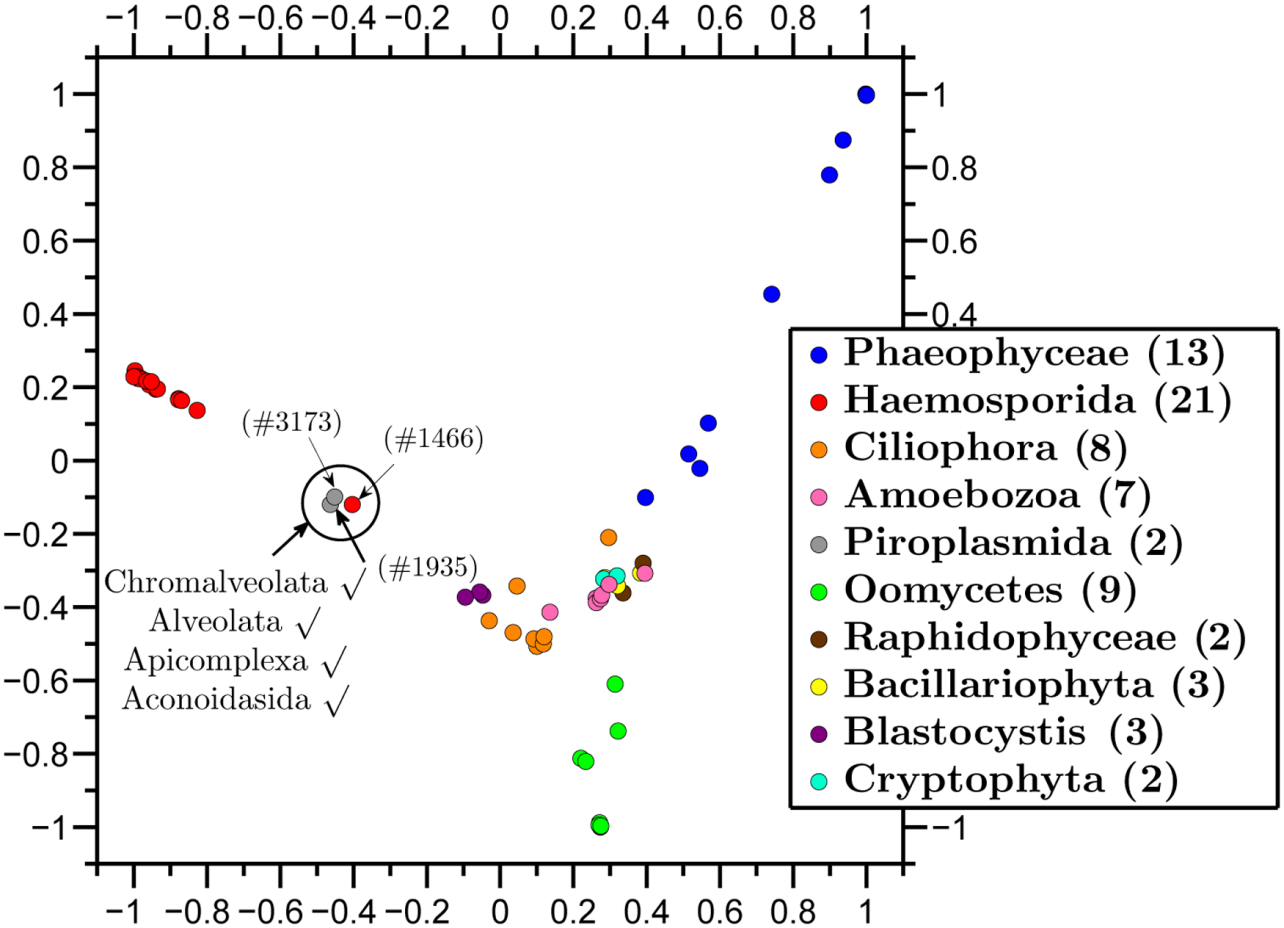
Molecular Distance Map of all represented species from (super)kingdom Protista and its orders. The total number of mtDNA sequences is 70, the average DSSIM distance is 0.8288, and the MDS *Stress-1* is 0.26. The sequence-point #1466 (red) is the unclassified *Haemoproteus* sp. jb1.JA27, #1935 (grey) is *Babesia bovis T2Bo*, and #3173 (grey) is *Theileria parva*. The annotation shows that all these three species belong to the same taxonomic groups, Chromalveolata, Alveolata, Apicomplexa, Aconoidasida, up to the order level.

We further applied our method to visualize the relationships among all represented species from the (super)kingdom Protista whose taxon, as defined in the legend of Fig. 3, had more than one representative. As expected, the maximum distance between pairs of sequences in this map was higher than the maximum distances for the other maps in this paper, all at lower taxonomic levels.

The most obvious outlier in Fig. 3 is *Haemoproteus* sp. jb1.JA27 (#1466), sequenced in [43] (see also [44]), and listed as an *unclassified* organism in the NCBI taxonomy. Note first that this sequence-point belongs to the same kingdom (Chromalveolata), superphylum (Alveolata), phylum (Apicomplexa), and class (Aconoidasida), as the other two species-points that appear grouped with it, *Babesia bovis* T2Bo (#1935), and *Theileria parva* (#3173). This indicates that its position is not fully anomalous. Moreover, as indicated by the high value of *Stress-1* for this figure, an inspection of DSSIM distances shows that this sequence-point may not be a true outlier, and its position may not be as striking in a higher-dimensional version of the Molecular Distance Map. Overall, this map shows that our method allows an exploration of diversity at the level of (super)kingdom, obtains good clustering of known subtaxonomic groups, while at the same time indicating a lack of genome sequence information and paucity of representation that complicates analyses for this fascinating taxonomic group.

**Fig 4:**
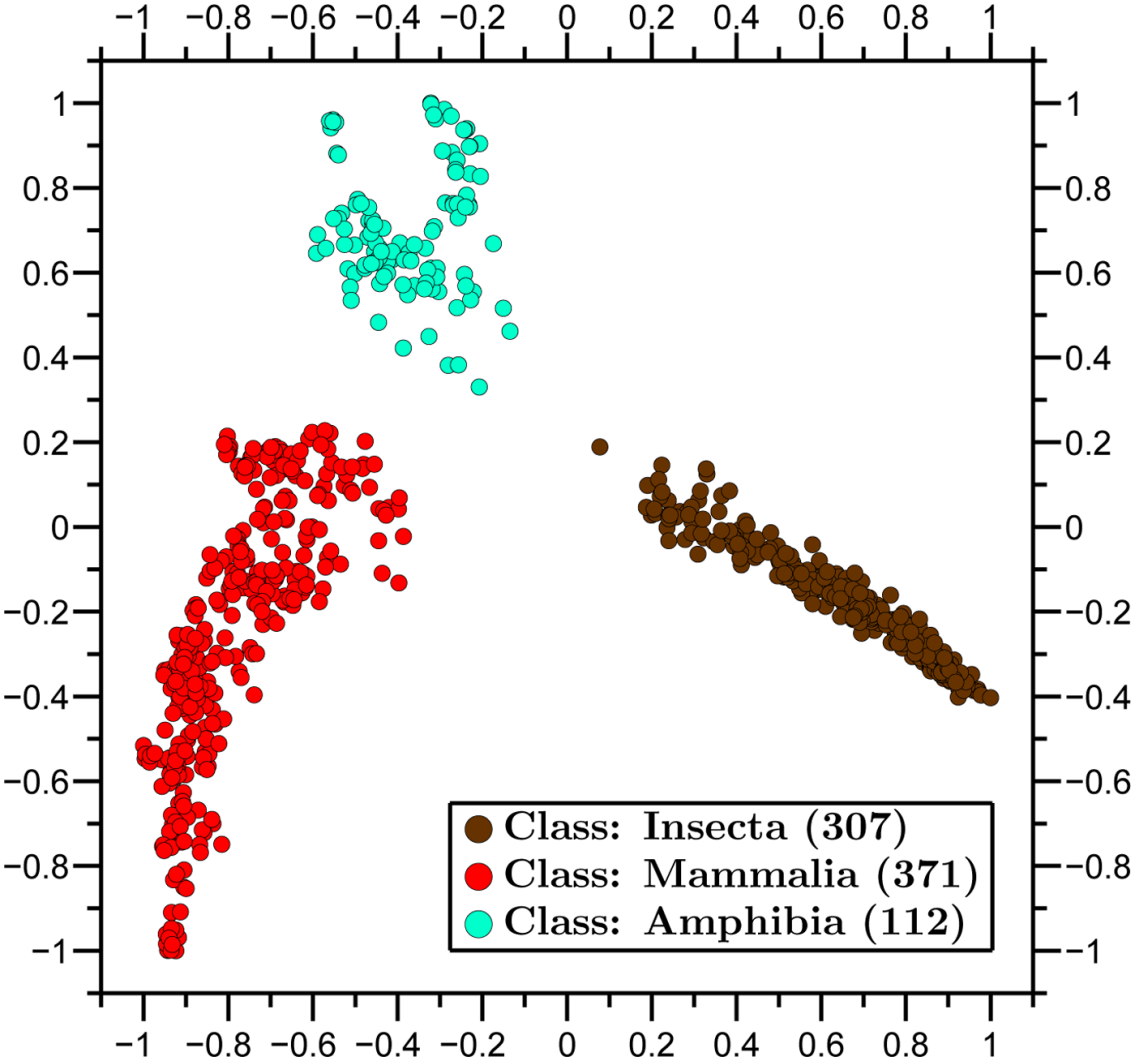
Molecular Distance Map of three classes: Amphibia, Insecta and Mammalia. The method successfully clusters taxonomic groups also at the Class level. Gaps and spaces in clusters, in this and other maps, may be due to sampling bias. A topic of further exploration would be to understand the cluster shapes and nature of the distribution of sequences in this figure. The total number of mtDNA sequences is 790, the average DSSIM distance is 0.8139, and the MDS *Stress-1* is 0.16.

**Fig 5:**
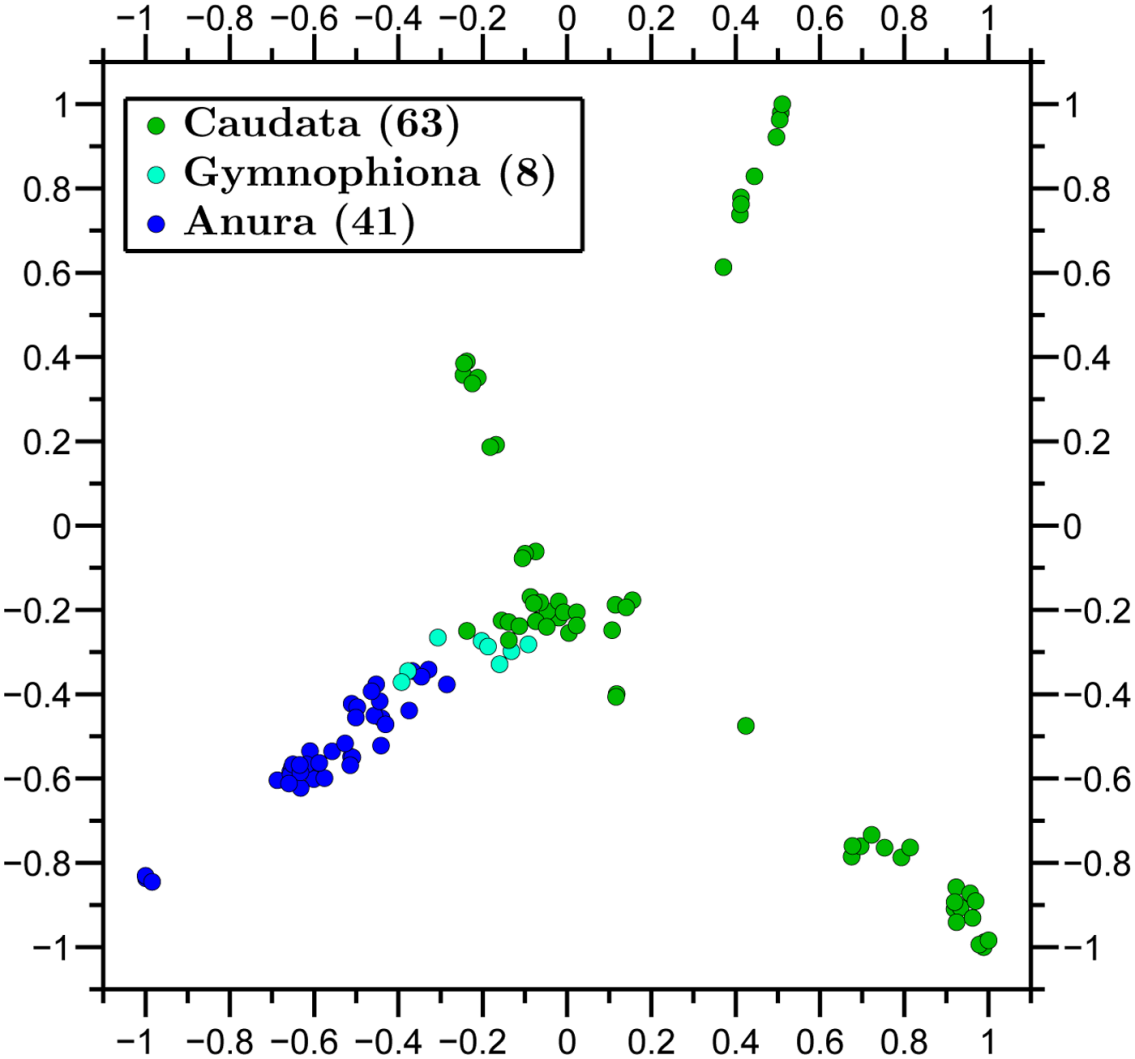
Molecular Distance Map of class Amphibia and three of its orders. The total number of mtDNA sequences is 112, the average DSSIM distance is 0.8445, and the MDS *Stress-1* is 0.18. Note that the shape of the amphibian cluster and the (*x*, *y*)-coordinates of sequence-points are different here from those in Fig. 4. This is because MDS outputs a map that aims to preserve pairwise distances between points, but not necessarily their absolute coordinates.

We then applied our method to visualize the relationships between all available complete mtDNA sequences from three classes, Amphibia, Insecta and Mammalia (Fig. 4), as well as to observe relationships within class Amphibia and three of its orders (Fig. 5).

A feature of MDS is that the points *p*_*i*_ are not unique. Indeed, one can translate or rotate a map without affecting the pairwise spatial distances *d*(*i*, *j*) = ∣∣*p*_*i*_ − *p*_*j*_∣∣. In addition, the obtained points in an MDS map may change coordinates when more data items are added to or removed from the dataset. This is because the output of the MDS aims to preserve only the pairwise spatial distances between points, and this can be achieved even when some of the points change their coordinates. In particular, the (*x*, *y*)-coordinates of a point representing the mtDNA sequence of an amphibian species in the Amphibia-Insecta-Mammalia map (Fig. 4) will not necessarily be the same as the (*x*, *y*)-coordinates of the same point when only amphibians are mapped (Fig. 5).

**Fig 6:**
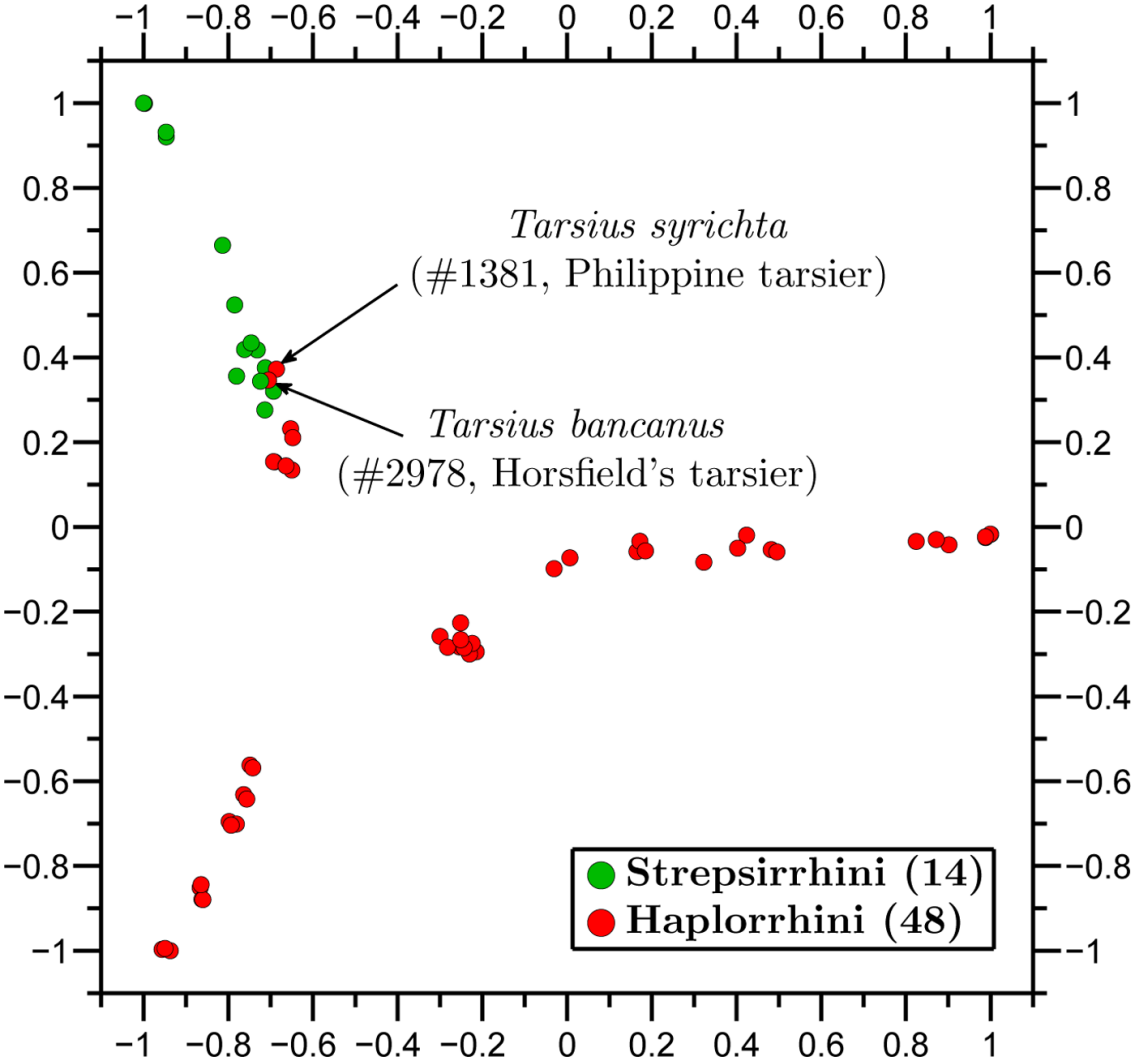
Molecular Distance Map of order Primates and its suborders: Haplorrhini (anthropoids and tarsiers), and Strepsirrhini (lemurs, lorises, etc.). The total number of mtDNA sequences is 62, the average DSSIM distance is 0.7733, and the MDS *Stress-1* is 0.19. The outliers are *Tarsius syrichta* #1381, and *Tarsius bancanus* #2978, whose placement within the order Primates has been subject of debate for over a century.

In general, Molecular Distance Maps are in good agreement with classical phylogenetic trees at all scales of taxonomic comparisons, see Fig. 5 with [45], and Fig. 6 with [46]. In addition, our approach may be able to weigh in on conflicts between taxonomic classifications based on morphological traits and those based on more recent molecular data, as in the case of tarsiers, discussed below.

Zooming in, we observed the relationships within an order, Primates, with its suborders (Fig. 6). Notably, two extinct species of the genus *Homo* are represented: *Homo sapiens neanderthalensis* and *Homo sapiens ssp. Denisova*. Primates can be classified into two groups, Haplorrhini (dry-nosed primates comprising anthropoids and tarsiers) and Strepsirrhini (wet-nosed primates including lemurs and lorises). Fig. 6 shows a clear separation of these suborders, with the top-left arm of the map comprising the Strepsirrhini. However, there are two Haplorrhini placed in the Strepsirrhini cluster, namely *Tarsius bancanus* (#2978, Horsfield’s tarsier) and *Tarsius syrichta* (#1381, Philippine tarsier). The phylogenetic placement of tarsiers within the order Primates has been controversial for over a century, [47]. According to [48], mitochondrial DNA evidence places tarsiiformes as a sister group to Strepsirrhini, while in contrast, [49] places tarsiers within Haplorrhini. In Fig. 6 the tarsiers are located within the Strepsirrhini cluster, thus agreeing with [48]. This may be partly because both this study and [48] used mitochondrial DNA, whose signature may be different from that of chromosomal DNA as seen in Fig. 1(a) and Fig. 1(b).

The DSSIM distances computed for all pairs of complete mtDNA sequences varied in range. The minimum distance was 0, between two pairs of identical mtDNA sequences. The first pair comprised the mtDNA of *Rhinomugil nasutus* (#98, shark mullet, length 16,974 bp) and *Moolgarda cunnesius* (#103, longarm mullet, length 16,974 bp). A base-to-base sequence comparison between these sequences (#98, NC_017897.1; #103, NC_017902.1) showed that the sequences were indeed identical. Subsequently, the sequence for species #103 was updated to a new version (NC_017902.2), on 7 March, 2013, and is now different from the sequence for species #98 (NC_017897.1). The second pair comprises the mtDNA sequences #1033 and #1034 (length 16,623 bp), generated by crossing female *Megalobrama amblycephala* with male *Xenocypris davidi* leading to the creation of both diploid (#1033) and triploid (#1034) nuclear genomes, [50], but identical mitochondrial genomes.

The maximum distance was found to be between *Pseudendoclonium akinetum* (#2656, a green alga, length 95,880) and *Candida subhashii* (#954, a yeast, length 29,795). Interestingly, the pair with the maximum distance Δ(#2656, #954) = 1.0033 featured neither the longest mitochondrial DNA sequence, with the darkest CGR (*Cucumis sativus*, #533, cucumber, length 1,555,935 bp), nor the shortest mitochondrial DNA sequence, with the lightest CGR (*Silene conica*, #440, sand catchfly, a plant, length 288 bp).

**Fig 7:**
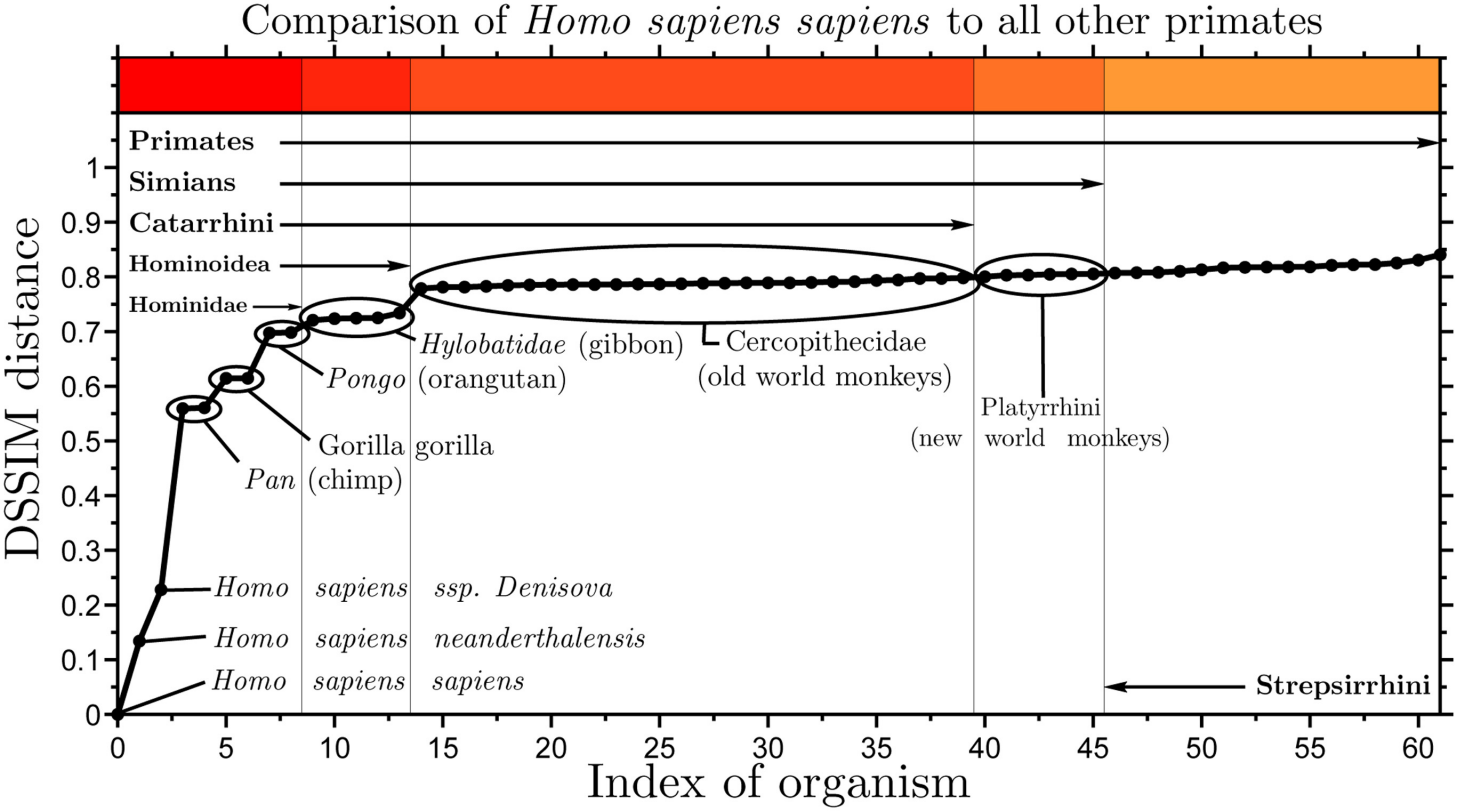
Graph of the DSSIM distances between the CGR images of *Homo sapiens sapiens* mtDNA and the CGR images of each of the 62 primate mitochondrial genomes (sorted by their distance from the human mtDNA). The distances are in accordance with established phylogenetic trees: The species with the smallest DSSIM distances from *Homo sapiens sapiens* are *Homo sapiens neanderthalensis*, *Home sapiens ssp. Denisova*, followed by the chimp.

An inspection of the distances between *Homo sapiens sapiens* and all the other primate mitochondrial genomes in the dataset showed that the minimum distance to *Homo sapiens sapiens* was Δ(#1321, #1720) = 0.1340, the distance to *Homo sapiens neanderthalensis* (#1720, Neanderthal), with the second smallest distance to it being Δ(#1321, #1052) = 0.2280, the distance to *Homo sapiens ssp. Denisova* (#1052, Denisovan). The third smallest distance was Δ(#1321, #3084) = 0.5591 to *Pan troglodytes* (#3084, chimp). Fig. 7 shows the graph of the distances between the *Homo sapiens sapiens* mtDNA and each of the primate mitochondrial genomes. With no exceptions, this graph is in full agreement with established phylogenetic trees, [46]. The largest distance between the *Homo sapiens sapiens* mtDNA and another mtDNA sequence in the dataset was 0.9957, the distance between *Homo sapiens sapiens* and *Cucumis sativus* (#533, cucumber, length 1,555,935 bp).

In addition to comparing real DNA sequences, this method can compare real DNA sequences to computer-generated sequences. As an example, we compared the mtDNA genome of *Homo sapiens sapiens* with one hundred artificial, computer-generated, DNA sequences of the same length and the same trinucleotide frequencies as the original. The average distance between these artificial sequences and the original human mitochondrial DNA is 0.8991. This indicates that all “human” artificial DNA sequences are more distant from the *Homo sapiens sapiens* mitochondrial genome than *Drosophila melanogaster* (#3120, fruit fly) mtDNA, with Δ(#3120, #1321) = 0.8572. This further implies that trinucleotide frequencies may not contain sufficient information to classify a genetic sequence, suggesting that Goldman’s claim [51] that “CGR gives no futher insight into the structure of the DNA sequence than is given by the dinucleotide and trinucleotide frequencies” may not hold in general.

The *Stress-1* values for all but one of the Molecular Distance Maps in this paper were in the “acceptable” range [0, 0.2], the exception being Fig. 3 with *Stress-1* equal to 0.26. However, note that *Stress-1* generally decreases with an increase in the map’s dimensionality, from two to three or to a higher number of dimensions. In addition, as suggested in [28], the *Stress-1* guidelines are not absolute: It is not always the case that only MDS representations with *Stress-1* under 0.2 are acceptable, nor that all MDS representations with *Stress-1* under 0.05 are good.

In all the calculations in this paper, we used the full mitochondrial sequences. Since the length of a sequence can influence the brightness of its CGR and thus its Molecular Distance Map coordinates, further analysis is needed to elucidate the effect of sequence length on the positions of sequence-points in a Molecular Distance Map. The choice of length of DNA sequences used may ultimately depend on the particular dataset and particular application.

We now discuss some limitations of the proposed method. Firstly, DSSIM is very effective at picking up subtle differences between images. For example, all vertebrate CGRs present the triangular fractal structure seen in the human mtDNA, and are visually very similar, as seen in Fig. 1(a) and Fig. 1(c). In spite of this, DSSIM is able to detect a range of differences that is sufficient for a good positioning of all 1,791 mtDNA sequences relative to each other. This being said, DSSIM may give too much weight to subtle differences, so that small and big differences in images produce distances that are numerically very close. This may be a useful feature for the analysis of datasets of closely related sequences. For large-scale taxonomic comparisons however, refinements of DSSIM or the use of other distances needs to be explored, that would space further apart the values of distances arising from small differences versus those arising from big-pattern differences between images.

Secondly, MDS always has some errors, in the sense that the spatial distance between two points does not always reflect the original distance in the distance matrix. For fine analyses, the placement of a sequence-point in a map has to be confirmed by checking the original distance matrix. Possible solutions include increasing the dimensionality of the maps to three-dimensional maps, which are still easily interpretable visually and have been shown in some cases to separate clusters which seemed incorrectly intermeshed in the two-dimensional version of the map. Other possibilities include a colour-scheme that would colour points with low stress-per-point differently from the ones with high stress-per-point, and thus alert the reader to the regions where discrepancies between the spatial distance and the original distance exist.

Thirdly, we note that the use of the particular distance measure (DSSIM) or particular scaling technique (classical MDS) does not mean that these are the optimal choices in all cases.

Lastly, as seen in Fig. 1(a) and Fig. 1(b), the genomic signature of mtDNA can be very different from that of nuclear DNA of the same species and care must be employed in choosing the dataset and interpreting the results.

### Conclusions

Our analysis suggests that the oligomer composition of mitochondrial DNA sequences can be a source of taxonomic information. These results are of interest both because of the large dataset considered (see, e.g., the correct grouping in taxonomic categories of 1,791 mitochondrial genomes in Fig. 2), and because this method circumvents the need for sequence similarity and extracts information from DNA sequences that normally would not be considered when using local, homology-based comparisons.

Potential applications of Molecular Distance Maps—when used on a dataset of genomic sequences, whether coding or non-coding, homologous or not homologous, of the same length or vastly different lengths—include identification of large evolutionary lineages, taxonomic classifications, species identification, as well as quantitative definitions of the notion of species and other taxa.

Possible extensions include generalizations of MDS, such as 3-dimensional MDS, for improved visualization, and the use of increased oligomer length (higher values of *k*) for comparisons of longer subsequences in case of whole chromosome or whole genome analyses. Lastly, it is worth mentioning that this method can be applied to analyzing sequences over other alphabets. For example binary sequences could be imaged using a square with vertices labelled 00, 01, 10, 11, and then DSSIM and MDS could be employed to compare and map them.
